# Cerebral Microbleeds, Hypertension, and Intracerebral Hemorrhage in Cerebral Autosomal-Dominant Arteriopathy with Subcortical Infarcts and Leukoencephalopathy

**DOI:** 10.3389/fneur.2017.00203

**Published:** 2017-05-15

**Authors:** Jung Seok Lee, KeunHyuk Ko, Jung-Hwan Oh, Joon Hyuk Park, Ho Kyu Lee, David Floriolli, Annlia Paganini-Hill, Mark Fisher

**Affiliations:** ^1^Department of Neurology, Jeju National University School of Medicine, Jeju City, South Korea; ^2^Department of Neurology, University of California Irvine School of Medicine, Irvine, CA, United States; ^3^Department of Psychiatry, Jeju National University School of Medicine, Jeju City, South Korea; ^4^Department of Radiology, Jeju National University School of Medicine, Jeju City, South Korea; ^5^Department of Radiological Sciences, University of California Irvine School of Medicine, Irvine, CA, United States; ^6^Department of Anatomy & Neurobiology, University of California Irvine School of Medicine, Irvine, CA, United States; ^7^Department of Pathology & Laboratory Medicine, University of California Irvine School of Medicine, Irvine, CA, United States

**Keywords:** CADASIL, microbleeds, hypertension, intracerebral hemorrhage, stroke

## Abstract

**Background:**

Cerebral autosomal-dominant arteriopathy with subcortical infarcts and leukoencephalopathy (CADASIL) is the most common genetic cause of stroke. In addition to ischemic stroke, CADASIL predisposes to development of cerebral microbleeds (CMB). CMB and hypertension are known to be associated with intracerebral hemorrhage (ICH). The purpose of this study was to analyze the relationships among CMB, hypertension, and ICH in CADASIL.

**Materials and methods:**

We enrolled 94 genetically confirmed CADASIL patients from 76 unrelated families at Jeju National University Hospital (Korea) between March 2012 and February 2015. We analyzed CMB presence, number, and distribution on susceptibility-weighted imaging MRI using the microbleed anatomical rating scale. Multiple logistic regression was used to determine factors associated with the presence of CMB and ICH.

**Results:**

CMB were observed in 62 patients (66%), median number of CMB per patient was 4 (range 0–121). Twenty-two ICHs were found in 16 patients (17%). There was incongruence between the most common site of CMB (thalamus) and that of ICH (basal ganglia). Hypertension was independently associated with the presence of CMB (multiple regression OR, 2.71; 95% CI 1.02–7.18, *p* < 0.05), and CMB ≥ 9 (highest third) was significantly associated with the presence of ICH (multiple regression OR = 9.50, 95% CI 1.08–83.71, *p* < 0.05).

**Conclusion:**

In this CADASIL sample, presence of hypertension was independently associated with CMB presence, and CMB burden was independently associated with ICH. Incongruence of sites for CMB and ICH is currently unexplained and requires further study.

## Introduction

Cerebral autosomal-dominant arteriopathy with subcortical infarcts and leukoencephalopathy (CADASIL) is the most common genetic cause of stroke. In addition to ischemic stroke, CADASIL predisposes to development of cerebral microbleeds (CMB) (1–3). CMB are well-defined MRI-demonstrable brain lesions consisting of tiny perivascular hemosiderin deposits, detectable using T2*-weighted gradient echo or susceptibility-weighted imaging (SWI) (4). CMB are associated with hypertension, cerebral amyloid angiopathy (CAA), and chronic kidney disease (5). CMB are also associated with intracerebral hemorrhage (ICH), which accounts for 20–30% of stroke in Asian countries such as Korea and Japan (6). Hypertension and CAA are the most common causes of ICH, and CAA is known to be associated with apolipoprotein E (ApoE)-ε4 genotype (7).

The presence of hemorrhagic phenomena in CADASIL creates clinical concerns, including potential use of antithrombotic treatments. In the current study, we investigated hemorrhagic complications of CADASIL. We analyzed the relationships among hypertension, CMB, and ICH, as well as the roles of other vascular factors including ApoE genotype. We hypothesized that hypertension and CMB would be independently associated with ICH, known to occur in 12–25% of East-Asian CADASIL patients (8–10). We also hypothesized that CMB would colocalize with ICH.

## Materials and Methods

We studied consecutive patients diagnosed with CADASIL by mutation of NOTCH3 gene (11) at Jeju National University Hospital’s Neurology Department (Korea) between March 2012 and February 2015. Asymptomatic as well as symptomatic patients were included in the study. This study was carried out in accordance with the recommendations of and approved by the Institutional Review Board of Jeju National University Hospital. All subjects gave written informed consent in accordance with the Declaration of Helsinki.

Symptomatic patients had been diagnosed with CADASIL based on CADASIL symptoms (ischemia or hemorrhagic episode, cognitive impairment, chronic headache, and/or seizure). Asymptomatic patients had at least one symptomatic family member. Vascular risk factors were recorded in all patients. Hypertension was defined as blood pressure >140/90 mm Hg on more than one occasion or use of antihypertensive agent. Diabetes mellitus was defined as fasting glucose level ≥126 mg/dl, PP2 test level ≥200 mg/dl, or use of anti-diabetes medication. Hypercholesterolemia was defined as total serum cholesterol level >240 mg/dl. In addition, a medication history, including use of platelet medications, was taken on the day of examination. ApoE genotype was determined in all patients (12).

All patients had one MRI study acquired on a 3 T scanner (Achieva, Philips Healthcare, Best, Holland) using a 32-channel array head coil. A volume isotropic TSE (turbo spin echo) acquisition technique was used for 3D FLAIR imaging. The parameters for 3D FLAIR imaging were as follows: TR/TE, 4,800/320 ms; TI, 1,650 ms; turbo factor, 240; spatial resolution, 1 mm × 1 mm × 1 mm; reconstructed resolution, 1 mm × 1 mm × 0.5 mm; and SENSE factor, 5. The acquisition time for 3D FLAIR was about 6 min 48 s. A 3D T1-weighted turbo field echo (TFE) acquisition technique was used for 3D T1-weighted imaging. The parameters for 3D T1 TFE were as follows: TR/TE, 8/4 ms; flip angle, 8°; spatial resolution, 1 mm × 1 mm × 1 mm; reconstructed resolution, 1 mm × 1 mm × 0.5 mm; and SENSE factor, 2: acquisition time, 5 min. SWI was performed for the evaluation of microbleeds. The detailed image parameters for SWI were as follows: flow-compensated three-dimensional gradient-echo sequence; TR/TE, 15/21 ms; flip angle, 15°; FOV, 210 mm × 210 mm; matrix, 280 × 280; section thickness, 2 mm; slab thickness, 150 mm; SENSE factor, 2; and total acquisition time, 2 min 51 s. Axial TSE T2-weighted imaging was acquired (TR/TE, 3,200/80 ms). In addition, head CT scans (Siemens) were performed at the time of symptomatic ICH.

One experienced neuroradiologist (Ho Kyu Lee), blinded to clinical data, reviewed all MRI and rated definite CMB presence, number, and distribution on SWI using the Microbleed Anatomical Rating Scale (MARS) (13). He read 50 brain MRI twice, 1 week apart, and the intrarater reliabilities (Kappa statistic, intraclass correlation coefficient) were calculated for presence, tertiles, and number of CMB. In MARS, definite CMB were defined as small, rounded or circular, well-defined hypodense lesions within brain parenchyma with clear margins ranging from 2 to 10 mm in size on T2*-weighted images, and locations of CMB were classified into deep, lobar, and infratentorial categories. CMB mimics were carefully excluded using all available images. ICH was defined as spontaneous non-traumatic bleeding into the brain parenchyma without secondary causes. It was diagnosed on the basis of neuroimaging (CT images at symptomatic ICH onset or MR images at examination including SWI, T1WI, T2WI, and FLAIR) by one neuroradiologist (Ho Kyu Lee) and confirmed by a second neuroradiologist (David Floriolli). Asymptomatic ICH was defined as MRI (including SWI, T1WI, T2WI, and FLAIR) documented hemorrhage without associated symptoms. Data were analyzed using SPSS statistical software (version 20.0). Variables in Table 1 (all dichotomous except age, education, SBP, and DBP) with a *p*-value < 0.2 on simple regression analysis were considered as potential independent variables to determine whether or not hypertension or other factors were associated with the presence of CMB and whether or not hypertension, CMB tertile, or other factors were associated with the presence of ICH. Stepwise backward logistic regression of variables was performed to obtain a final model of significant (*p* < 0.05) independent variables. CMB count, not normally distributed, was categorized according to tertiles and an ordinal logistic regression was also performed.

**Table 1:** **Clinical characteristics of 94 patients with CADASIL**.

	Mean ± SD, median
Age, years	62.6 ± 12.5, 64.0
Education, years	9.3 ± 0.6, 12.0
Systolic blood pressure (mmHg)	122.8 ± 12.4, 123.0
Diastolic blood pressure (mmHg)	75.1 ± 9.7, 74.5
Number of CMB	9.7 ± 17.5, 4.0

	***n* (%)**

Males	52 (55)
ApoE ε4	30 (32)
Hypertension	50 (53)
Diabetes mellitus	16 (17)
Hypercholesterolemia	24 (26)
Atrial fibrillation	3 (3)
Platelet medication use	60 (64)
Anticoagulant use	2 (2)
Ever-smoking	36 (37)
CMB	62 (66)
Intracerebral hemorrhage	16 (17)

*CADASIL, cerebral autosomal dominant arteriopathy with subcortical infarcts and leukoencephalopathy; ApoE, apolipoprotein E; CMB, cerebral microbleeds*.

## Results

We studied 94 patients with genetically confirmed CADASIL, excluding three patients who could not complete MRI. These patients were from 76 families, and 89 patients (95%) had a R544C mutation, followed by R578C in 2 patients (2%), R75P in 2 patients (2%), and C452A in 1 patient (1%). Clinical characteristics of the 94 patients are summarized in Table 1; 52 patients were men (55%) and mean ± SD age of the patients was 62.6 ± 12.5 years (range 34–90). Hypertension was present in 50 patients (53%). The ApoE genotype frequencies were ε2/ε2 1 (1%); ε2/ε3 8 (9%); ε2/ε4 2 (2%); ε3/ε3 55 (59%); ε3/ε4 27 (29%); and ε4/ε4 1 (1%). Platelet medications included aspirin (*n* = 16), clopidogrel (*n* = 25), cilostazol (*n* = 14), triflusal (*n* = 2), combination of aspirin and clopidogrel (*n* = 1), and combination of aspirin and cilostazol (*n* = 2).

**Figure 1:**
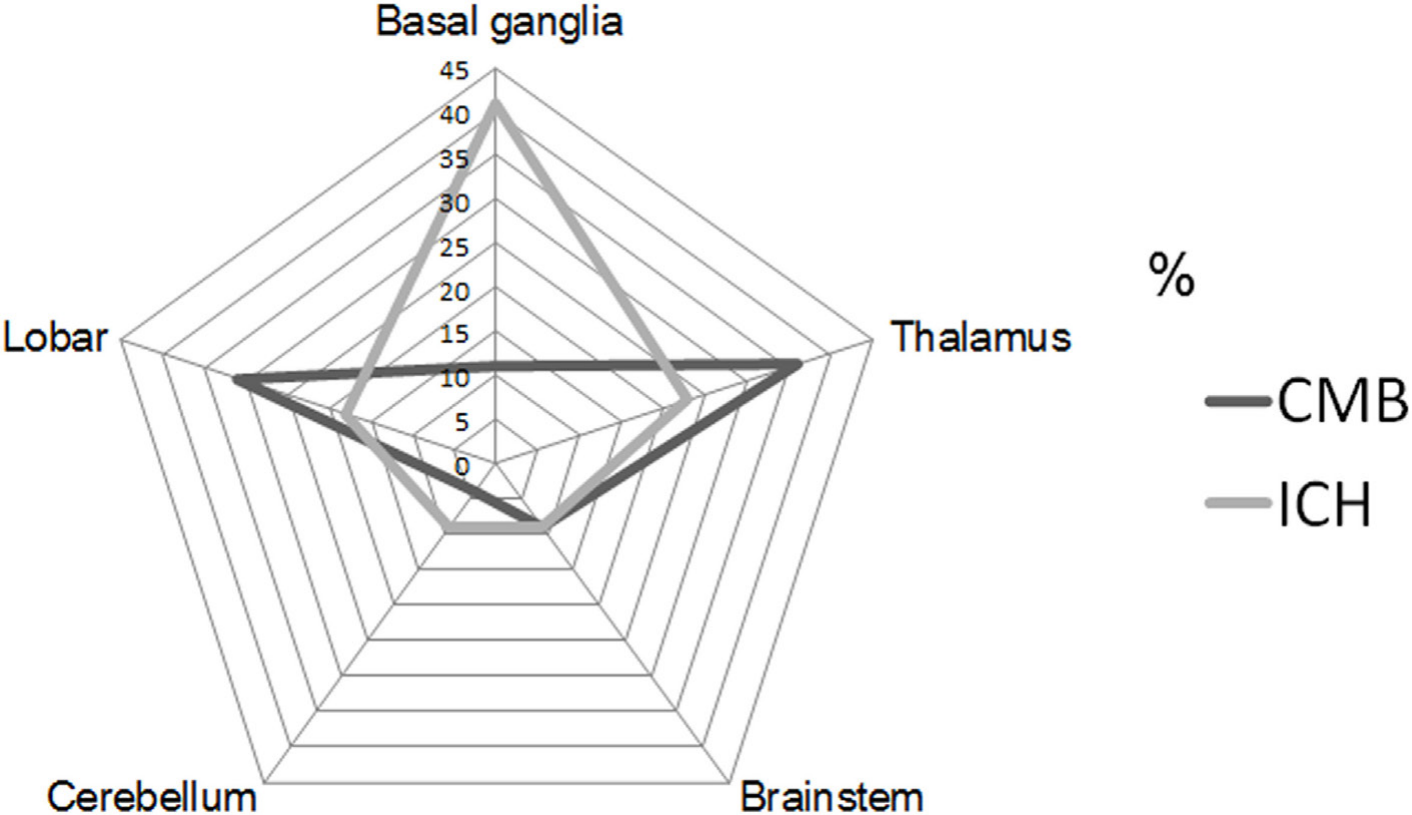
**Proportion of cerebral microbleeds (CMB) and intracerebral hemorrhage (ICH) by each location of brain**. CMB radar plots (black line) show the most common site was thalamus (36%). ICH radar plots (gray line) show that the most common site was basal ganglia (41%).

**Figure 2:**
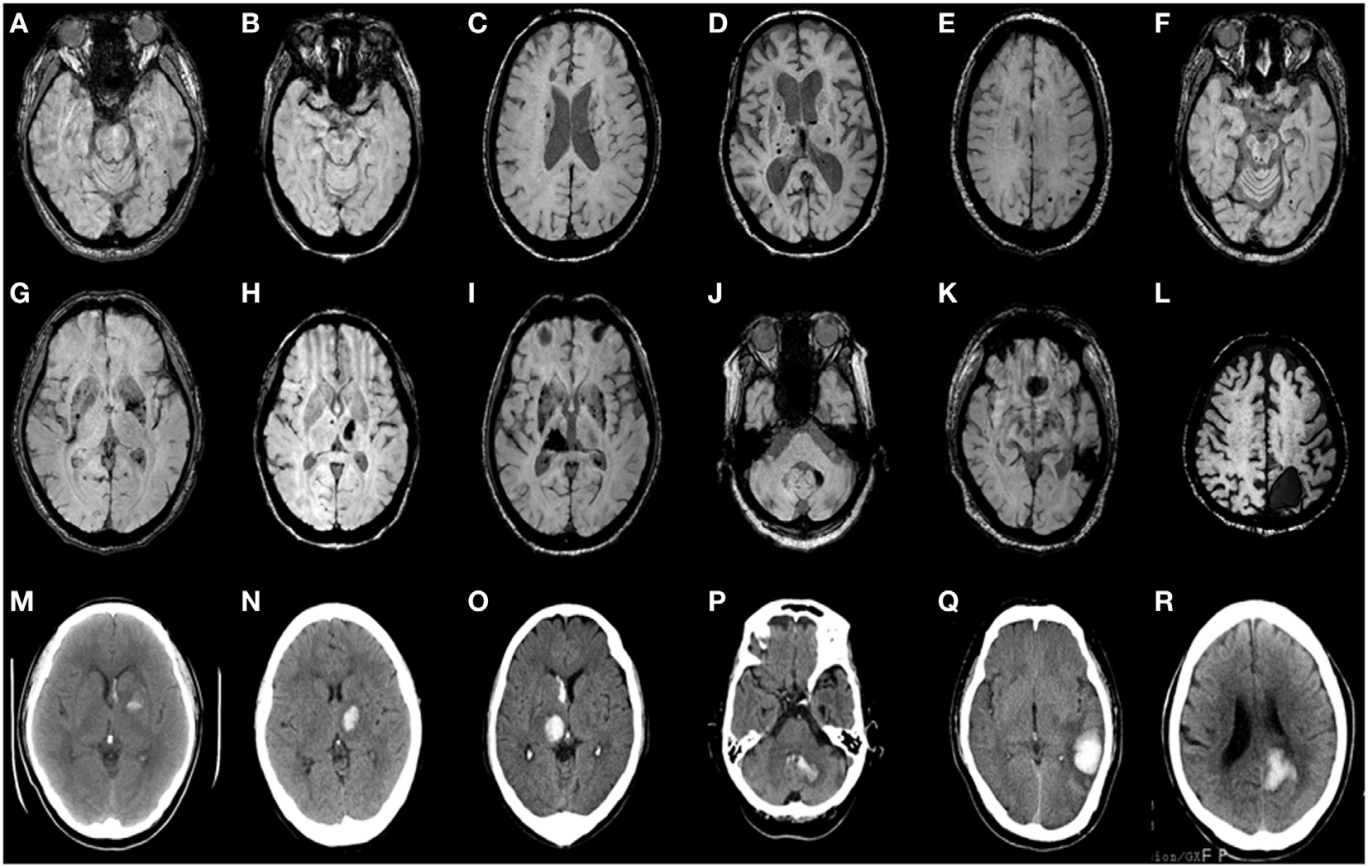
**Axial brain CT and matching susceptibility-weighted images (SWI) of six CADASIL patients with intracerebral hemorrhage (ICH) (A–R)**. SWI **(A–F)** shows cerebral microbleeds without ICH. SWI **(G–L)** and matching unenhanced brain CT **(M–R)** demonstrate acute ICH with or without intraventricular hemorrhage. Panels **(A,G,M)** are patient 1. **(B,H,N)** are patient 2. **(C,I,O)** are patient 3. **(D,J,P)** are patient 4. **(E,K,Q)** are patient 5. **(F,L,R)** are patient 6.

Intrarater reliabilities for CMB presence and number were excellent (Kappa statistic = 0.94 for presence and 0.97 for tertiles; intraclass correlation coefficient for number = 0.97, 95% CI 0.95–0.98). CMB were detected in 66% (62 out of 94). The number of CMB per patient ranged from 0 to 121 (median 4.0). Tertiles for CMB count gave: lowest third 0 CMB, middle third 1–8 CMB, and highest third ≥9 CMB. Among 958 CMB, 341 (36%) were located in the thalamus, 298 (31%) were lobar, 106 (11%) in the basal ganglia, 83 (9%) in the brainstem, 42 (4%) in cerebellum, 39 (4%) in deep periventricular white matter, 28 (3%) in internal capsule, and 21 (2%) in external capsule. Among the patients with CMB, 3% had strictly lobar CMB, 15% strictly deep CMB, and 2% strictly infratentorial CMB, whereas 80% had mixed CMB. Twenty-two ICHs were found in 16 patients (17%). Among 22 ICH, 9 (41%) were located in the basal ganglia, 5 (23%) in the thalamus, 4 (18%) in the lobar, 2 (9%) in the cerebellum, and 2 (9%) in pons (Figures 1 and 2). Four ICH were asymptomatic in four patients. The site of asymptomatic ICH was pons (*n* = 2), basal ganglia (*n* = 1), and lobar area (*n* = 1). Nine of 22 ICH (41%) had CMB in the same location as the ICH; 3 of 9 ICH in basal ganglia (33%) had CMB in the same location (Figures 1 and 2).

Age (continuous), ApoE genotype (ApoE-ε4 vs non-ApoE-ε4), hypertension, and platelet medication use were associated (*p* < 0.2) on simple logistic regression analysis with the presence of CMB (Table 2) and were, therefore, included as potential independent variables in the multiple logistic regression analysis for CMB (Table 3). Hypertension was independently associated with the presence of CMB (multiple regression OR, 2.71; 95% CI, 1.02–7.18; *p* = 0.045). Although platelet medication use was related to the presence of any CMB in simple logistic regression analysis (OR, 3.00; 95% CI, 1.23–7.31; *p* = 0.016), it was not significantly related in the final multiple logistic regression model. Age was independently associated with the presence of CMB (multiple regression OR, 1.06; 95% CI, 1.02–1.11; *p* = 0.006). Ordinal logistic regression of CMB tertile gave similar results: only hypertension (*p* = 0.0005) and age (*p* = 0.03) were significantly associated with CMB tertile. However, age was not associated with the presence of ICH in simple logistic regression analysis (*p* = 0.57). The highest CMB count group (≥9) was significantly associated with the presence of ICH (multiple regression OR = 9.50; 95% CI, 1.08–83.71; *p* = 0.043) (Table 3). Hypertension was related to the presence of ICH in simple logistic regression analysis (OR, 4.80; 95% CI, 1.27–18.19; *p* = 0.021) (Table 2), but not significantly related in the final multiple logistic regression model (*p* = 0.12).

**Table 2:** **Simple logistic regression odds ratios and 95% confidence intervals for presence of CMB and for ICH in 94 patients with CADASIL**.^†^

**CMB**	
Age	1.07 (1.03–1.17)**
APOE-ε4	2.11 (0.79–5.63)
Hypertension	4.00 (1.61–9.94)**
Platelet medication use	3.00 (1.23–7.31)*
**ICH**	
Age	1.01 (0.97–1.06)
APOE-ε4	2.55 (0.85–7.63)
Hypertension	4.80 (1.27–18.2)*
Platelet medication use	0.68 (0.23–2.03)
**CMB burden (tertiles)**	
I (CMB = 0)	1.00 (reference)
II (CMB = 1–8)	6.20 (0.68–56.6)
III (CMB ≥ 9)	14.09 (1.68–118)*

*CADASIL, cerebral autosomal dominant arteriopathy with subcortical infarcts and leukoencephalopathy; APOE-ε4, apolipoprotein E-ε4; CMB, cerebral microbleeds; ICH, intracerebral hemorrhage*.

*^†^Only variables with p < 0.2 are presented in the table*.

*p < 0.05, **p < 0.01.

**Table 3:** **Multiple logistic regression odds ratios and 95% confidence intervals for significant factors relating to the presence of CMB and ICH in 94 patients with CADASIL**.

**CMB**	
Age	1.06 (1.02–1.11)**
Hypertension	2.71 (1.02–7.18)*
**ICH**	
**CMB burden (tertiles)**	
I (CMB = 0)	1.00 (reference)
II (CMB = 1–8)	5.03 (0.53–47.1)
III (CMB ≥ 9)	9.50 (1.08–83.7)*

*CADASIL, cerebral autosomal dominant arteriopathy with subcortical infarcts and leukoencephalopathy; CMB, cerebral microbleeds; ICH, intracerebral hemorrhage*.

*p < 0.05, **p < 0.0.1.

## Discussion

We found that hypertension and age were independently associated with the presence of CMB in patients with CADASIL. We also found that the highest CMB count group (≥9) was independently associated with the presence of ICH. In addition, there was a mismatch between the most common site of CMB and ICH.

We observed that hypertension was independently associated with the presence of CMB (*p* = 0.045). This is in line with earlier studies (14, 15). In this study, we used MARS and SWI, which are known to increase the number and presence of CMB detected (16, 17). We observed excellent intrarater reliability for number of CMB, and prior work has shown excellent inter-rater reliability for CMB on SWI using MARS (18). Also, our results are consistent with a recent meta-analysis showing significant correlation between hypertension and CMB in patients with stroke and in a healthy population (19).

In the present study, we show that age was associated with the presence of CMB. Recent studies of the relationship between age and presence of CMB in CADASIL have produced conflicting results (2, 3, 14, 15). However, pathological analysis has shown that cerebral microhemorrhages are common in aging brain, regardless of presence of hypertension or CAA (20). Moreover, CMB increases with age for all locations in a general population (21), and age may be compounded by important vascular comorbidities for development of CMB (22, 23). Both CADASIL and hypertension have important effects on arteriolar function, with both processes targeting vascular smooth muscle cells (5).

We demonstrated that 17% of patients had primary ICH (*n* = 16); all had R544C mutation, and 14 had history of hypertension. In a Taiwanese series of 112 CADASIL patients in which R544C accounted for 71% of the mutation, the occurrence of ICH did not differ between R544C mutation group and non-R544C mutation group (9). However, unlike East-Asian CADASIL patients, ICH has rarely been reported in Caucasian CADASIL patients (24). Recent work described four unrelated Caucasian CADASIL patients with ICH (25), all of whom had history of hypertension. Nine Caucasian CADASIL cases with ICH have been reported, and all showed various NOTCH3 mutations (25). Therefore, it is unclear whether ICH occurrence in CADASIL patients was due to specific NOTCH3 mutation.

We found that CMB burden (≥9) was independently associated with the presence of ICH (*p* = 0.043). CMB may predict subsequent recurrent ICH in patients with previous lobar ICH (4, 18, 26). In addition, recent meta-studies have shown that CMB burden (≥10) on pretreatment MRI increases the risk of post-thrombolysis ICH during IV thrombolysis (27). Our findings are consistent with these observations suggesting that CMB burden predisposes to ICH.

There was a mismatch between the most common site of CMB and ICH in CADASIL in our study. The most common site of CMB was thalamus, followed by lobar, and basal ganglia. However, the most common site of ICH was basal ganglia, followed by thalamus, and lobar area. Because the presence of ICH may mask the presence of CMB, the topographic incongruence of CMB and ICH requires careful interpretation. However, the majority of the patients with CMB (47/62, 76%) had no ICH. The explanation for this mismatch is unclear. We recently proposed a “two site” model of CMB and ICH, in which the specific vascular sites for development of CMB and ICH are different (5). In both CADASIL and hypertension, (arteriolar) smooth muscle cells and blood–brain barrier are targeted; the former has been suggested as site for ICH and the latter a site for CMB (5). While the findings of the current study are consistent with this model, further mechanistic investigations are needed to confirm the observation.

Specific strengths of this study include the relatively large number of homogeneous CADASIL population (mostly R544C mutation), inclusion of ApoE genotype, and application of advanced MRI (including SWI). Our study has several limitations. First, the R544C mutation was predominant; however, genotype–phenotype differences in CADASIL are not known (28). Second, this was a cross-sectional study, and prospective studies are needed to elucidate our findings. Third, we limited our observations to hemorrhagic features and did not investigate other manifestations of small vessel disease such as white matter hyperintensities and small deep infarctions. Fourth, we did not compare our findings to those of a non-CADASIL (e.g., CAA) or a non-Asian cohort, so we cannot address whether our findings are unique for our population group. Fifth, as some asymptomatic patients were likely not identified, selection bias remains a possibility. Finally, while there was a topographical mismatch between CMB and ICH, presence of ICH may obscure presence of CMB.

Our results provide insights into interactions of CMB, hypertension, and ICH in patients with CADASIL. These findings are consistent with a model of hemorrhagic complications in CADASIL in which hypertension predisposes to CMB, which in turn predisposes to ICH. However, this scenario requires confirmation in longitudinal clinical investigations. The topographic incongruence of CMB and ICH is currently unexplained, but is consistent with a model of differing vascular sites for CMB and ICH in CADASIL. Overall, these findings indicate that interactions between CMB, hypertension, and ICH in CADASIL are complex and will benefit from intense mechanistic investigations.

## Author Contributions

Conception and design of the study: JL and MF. Acquisition of data: JL, KK, J-HO, and JP. Analysis of data: JL, HL, DF, AP-H, and MF. Drafting a significant portion of the manuscript: JL and MF.

## Conflict of Interest Statement

The authors declare that the research was conducted in the absence of any commercial or financial relationships that could be construed as a potential conflict of interest.
